# Vitamin D3 reduces hippocampal NR2A and anxiety in nicotine withdrawal mice

**DOI:** 10.1515/tnsci-2020-0166

**Published:** 2021-06-11

**Authors:** Bingxue Wu, Xinrong Tao, Chuanlin Liu, Huaixu Li, Tao Jiang, Zijun Chen, Qi Wang, Fei Liu, Min Mu, Zhaoyan Chen

**Affiliations:** School of Medicine, Anhui University of Science and Technology, Anhui Huainan 232001, China; Key Laboratory of Industrial Dust Purification and Occupational Health of the Ministry of Education, Anhui University of Science and Technology, Anhui Huainan 232001, China; Key Laboratory of Industrial Dust Deep Reduction and Occupational Health and Safety, Anhui Higher Education Institutes, Anhui University of Science and Technology, Anhui Huainan 232001, China; Anhui Province Engineering Laboratory of Occupational Health and Safety, Anhui University of Science and Technology, Anhui Huainan 232001, China; The First Affiliated Hospital of Anhui University of Science and Technology, Anhui Huainan 232001, China

**Keywords:** nicotine withdrawal, anxiety-like behavior, vitamin D3, NR2A, hippocampus

## Abstract

Nicotine withdrawal symptoms, mainly anxiety, cause high level of relapse rate after quitting smoking. Vitamin D supplementation has shown its potential for the prevention and treatment of anxiety disorders; however, neurobiological studies about the effect of vitamin D on nicotine withdrawal-induced anxiety are limited. To investigate the effect and molecular mechanism of vitamin D3 supplement by dietary on anxiety-like behavior during nicotine withdrawal, male C57/BL6 mice were divided into four groups: vehicle, nicotine only, vitamin D3 only, and nicotine plus vitamin D3. Mice were administrated with nicotine in drinking water (200 µg/mL), and vitamin D3 in feed for 6 weeks. During nicotine withdrawal, vitamin D3-treated mice showed significantly less anxiety-like behavior by an open-field test and marble buried test that performed an increase in the duration of the central zone and a decrease buried marble, respectively. Moreover, vitamin D3 supplementation attenuated the hippocampal NR2A expression on both protein and mRNA levels in nicotine and vitamin D3-treated mice. Our data showed that dietary supplementation with vitamin D3 ameliorated nicotine withdrawal-induced anxiety, which may be related to downregulation of NR2A expression in hippocampus. Vitamin D3 may provide a new dietary intervention with the easy access for smoking cessation.

## 1 Introduction

Tobacco kills more than 8 million people per year around the world [1]. There are 26.6% (or 308 million) of Chinese people aged 15 and above smoke tobacco according to the 2018 Chinese adult tobacco survey. Among them, 90% of smokers have the idea of quitting smoking, but only 20.1% of them succeed because of the lack of effective treatments for smoking cessation [2]. Nicotine with addictive nature is one of the major components in cigarettes. It is also a key mediator of negative effects of smoking. Nicotine withdrawal symptoms, the main characteristics of anxiety and depression after quitting smoking, are the key cause of high relapse rate [3]. Available interventions for smoking cessation, such as pharmacological treatments, behavioral or psychological interventions including physical training, and smoking cessation hotline, can only be helpful temporally. Thereby the relapse rate is still high [2]. Thus, looking for a simple, safe, and inexpensive supplementation to ease the nicotine withdrawal symptoms experienced by people when they quit smoking is essential for continuous abstinence from smoking.

In recent years, accumulative evidences have shown that hippocampal synaptic plasticity affect stress, emotions, and memory [4,5]. *N*-Methyl-d-aspartate (NMDA) receptor and downstream signaling regulate anxiety-like behavior, depression, and hippocampal-related spatial memory [5,6]. After NMDA receptor gene was knocked out in granule cell of the hippocampal dentate gyrus, long-term potentiation was damaged severely and spatial memory was impaired, but the anxiety-like behaviors were ameliorated. This suggests that the hippocampus not only played an important role in spatial memory, but the ventral hippocampus might also be involved in the regulation of anxiety-like behavior [7]. Moreover, NMDA receptors were overexpressed after nicotine withdrawal followed by anxiety-like behaviors [8].

NR2A (or GRIN2A, GluN2A), a subunit of the NMDA receptors, also one of the vitamin D receptor-regulated gene set, plays an indispensable role in the function of NMDA [29]. In addition, the activation of NMDA receptor channels and synaptic plasticity are closely related to the inward Ca^2+^ current [9]. Vitamin D can downregulate the expression of L-type calcium channels and reduce the concentration of extracellular Ca^2+^ in hippocampal neurons; at the same time, it has neuroprotective effect and can prevent excitotoxicity injury caused by overexpression of NMDA receptors [10]. Vitamin D3 has the highest biological metabolism rate and the highest bioavailability among vitamin D. This study aims to investigate the effect of vitamin D3 on nicotine withdrawal-induced anxiety-like behavior in mice, and explore its underlying mechanism, finding new ways to help the nicotine dependent to quit.

## 2 Methods

### 2.1 Animals

Adult C57BL/6 male mice (9–10 weeks old, 18 ± 2 g) were purchased from the Changzhou Cavion Experimental Animal Co, Ltd. (license number SCXY (Su) 2011-0003). Mice were habituated to a vivarium supported with a standard 12 h light–dark cycle (lights on at 07:00 AM), with regular temperature and humidity (22℃ and 50%, respectively). Meanwhile, they were free to food and water and caged separately.

### 2.2 Chemicals and reagents

Nicotine in drinking water (Sigma, N-3876; 200 μg/mL) contains 1% saccharin (Shyuanye, 128-44-9). Ordinary feed: composed of corn meal, flour, wheat husk, vitamins, soybean meal, oil residue, fish meal, cod liver oil, stone powder, and salt. Vitamin D3 special feed: vitamin D3 (10,000 U/Kg) was added to ordinary feed. Previously, vitamin D3 supplementation with 10,000 IU QD improved the anxiety and depression scores in patients with Crohn’s Disease [11]. α7 nAChR (a subunit of neuronal nicotinic acetylcholine receptors) primary antibody (ab23832, 1:1,000), and Goat anti-rabbit IgG H&L (ab6721, 1:10,000) which was the secondary antibody came from Abcam (Cambridge, United Kingdom). NR2A primary antibody (19953-1-AP, 1:800) and GAPDH primary antibody (10494-1-AP, 1:10,000) were purchased from proteintech. 5× All-In-One RT MasterMix kit (Bioland Scientific LLC, FS01-01) and GoTaq^®^ qPCR Master Mix (promega, A6020) were used for cDNA synthesis. The gene-specific primers, NR2A, α7 nAChR, and GAPDH, were purchased from Sangon Biotech (Shanghai, China).

### 2.3 Nicotine exposure

C57/BL6 male mice were randomly divided into four groups by random digital table method: (1) vehicle group (Veh group, *n* = 16): ordinary feed, drinking water (1% sodium saccharin); (2) vitamin D3 control group (VD group, *n* = 8): vitamin D3 special feed, drinking water (1% sodium saccharin); (3) nicotine group (Nic group, *n* = 16): ordinary feed, nicotine (200 μg/mL) dissolved in 1% sodium saccharin; (4) vitamin D3_+_ nicotine group (NV group, *n* = 16): vitamin D3 special feed, nicotine (200 μg/mL) dissolved in 1% sodium saccharin. The duration of all exposure was 42 days. The dose and duration of nicotine administration were selected based on previous studies [12,14]. The drinking flowing in each group was changed daily, and food intake was measured every 3 days. Body weight was measured every other day. Nicotine exposure can slow down the rate of weight gain [13].

### 2.4 Open field test (OFT)

Spontaneous nicotine withdrawal was induced as described in the literature [12,14]. Withdrawal behavior in mice as early as 24 h post last drink within the first 48 h is evident [15]. We replaced the drinks in nicotine-treated mice in Nic and NV group with 1% sodium saccharin water bottles. Anxiety-like behaviors were measured within 24–48 h after nicotine bottle replacement. The OFT is used to access locomotor and anxiety-like behaviors [16]. Briefly, the experiment was accomplished in a quiet environment, and mice were acclimatized to the behavioral recording room for 3 days, 60 min a day. Subsequently, they were smoothly put in a center of an open-field Plexiglas clear chamber (30 cm × 30 cm × 35 cm, Anhui Huaibei Zhenghua Experimental Instrument Co., Ltd.) and granted to move freely for 30 min. The ground zone of the box was divided into the peripheral zone (area within 7.5 cm away from the edge) and central zone (the rest area). Behavior in the open field arena was tracked using an overhead video camera, which linked EthoVision XT 5.1 detection system (Noldus Information Technology, The Netherlands). Clean all chambers fully with 10% alcohol before proceeding to the next test. The anxiety-like behaviors were measured by the duration in the center area, and basal exploration activity was appraised by the total distance traveled (cm) and immobility (s). The visual figure, movement track over the duration of the testing, was used to show the difference in the traveled distance in the center square. Open-field testing was carried out followed by marble burying test (MBT) with over 30 min interval.

### 2.5 Marble burying test

The MBT box (30 cm × 15 cm × 35 cm) was covered with 5 cm thick bedding, and 20 glass beads (1.4 cm in diameter) were evenly placed on the corncob bedding. The glass beads were more than 1 cm from the edge of the box. Mice were placed in its test box and freely explored for 30 min. The number of buried marbles with bedding (to 2/3 their depth) was used as the observation index. Moreover, anxiety-related behavior was evaluated by the buried numbers during the trial.

### 2.6 Cotinine and 25(OH)D3 measurements

One day before nicotine withdrawal (day 41), ∼300 µL of blood sample was collected from the mouse. The blood samples were kept stable and congealed at room temperature for 1 h. Then, the samples were centrifuged at 3,000 rpm for 15 min at 4°C, and the resulting supernatant were collected and kept at −80℃. The content of 25(OH)D3 and cotinine in mouse serum were determined by enzyme linked immunosorbent assay (ELISA) kits according to the instructions. Commercially available mouse 25(OH)D3 ELISA kit (SBJ-M0973, SenBeiJia Biological Technology Co., Ltd., Nanjing, China) and mouse cotinine ELISA kit (JL20440, Jianglaibio, Shanghai, China) were purchased. 25(OH)D3 is the major storage form of vitamin D in the body. Cotinine is the main product after the primary metabolism of nicotine. When the concentration of cotinine in the blood exceeds 300 ng/mL, it is equivalent to the level of nicotine addiction [17].

### 2.7 Tissue extraction

Mice were anesthetized with 10% chloral hydrate 2 mL/kg, minutes after the end of the behavioral experiments. After cardiac perfusion with cold 0.9% saline, the brain was quickly decapitated. The hippocampus tissue was collected immediately with RIPA lysis buffer (P0013B, Beyotime, Shanghai, China) containing PMSF and phosphatase inhibitor on ice for 15 min. One side of hippocampus tissue was used for QPCR, and the other one for western blot (WB).

### 2.8 Quantitative reverse transcription PCR (RT-qPCR)

Mouse hippocampus were collected on ice and stored at −80℃. Total RNA of them was extracted by Trizol. Sensitive first-strand cDNA synthesis kit for gene expression analysis was used for cDNA synthetization. Real-time qPCR was carried out on the equipment (ABI, QuantStudio3) using the two-step PCR reaction conditions: first 95℃ 10 min pre-denaturation, then 95℃ 10 s, and 58℃ 60 s for amplification. Forty cycles were repeated to obtain the Ct value. 2^(−ΔΔCT)^ method was applied to calculate the relative expression of the target gene. GAPDH was served as reference gene. The primer sequences are shown in Table 1.

**Table 1:** Primers used for RT-PCR analysis

Target gene	Primer sequence
NR2A forward 5′–3′	AGACCTTAGCAGGCCCTCTC
NR2A reverse 3′–5′	CTCTTGCTGTCCTCCAGACC
α7 nAChR forward 5′–3′	TACTTCTCCCTGAGCCTCCT
α7 nAChR reverse 3′–5′	GTTGGTGTGGAATGTGGCAT
GAPDH forward 5′–3′	GTGGGTGCAGCGAACTTTAT
GAPDH reverse 3′–5′	CACTGAGCATCTCCCTCACA

### 2.9 WB analysis

Bicinchoninic acid Protein Assay kit (P000s9, Beyotime, Shanghai, China) was used to detect the protein concentration. Equal content of protein (35 μg) was detached by 10% SDS PAGE (electrophoresis, 100 V running to the end), and transferred to a polyvinylidene fluoride membrane (electroporation, 200 mA 4 h). The membrane was blocked with 5% skim milk in TBST buffer for 2 h before being incubated with primary antibody, α7 nAChR (1:1,000), GAPDH (1:10,000), NR2A (1:800), at 4°C overnight. The goat anti-rabbit IgG-HRP secondary antibody (1:10,000) was added and incubated for 40 min after the membrane was washed thoroughly with TBST five times. The WBs were visualized with Chemiluminescent HRP Substrate (P90720, Millipore Corporation, Burlington, MA) and measured with gel imaging system [Molecular Imager ChemiDocTM XRS+ analysis system (BioRad Co., Hercules, CA)]. After scanning, the image was analyzed by Image J. GAPDH was served as reference protein.

### 2.10 Statistical analysis

Data were analyzed using one-way ANOVA followed by Bonferroni *post hoc* tests as indicated. *P*-values <0.05 were considered statistically significant. All data were expressed as mean ± SEM.


**Ethical approval:** All procedures were conducted in accordance with the guidelines as described in the National Institutes of Health’s Guide for the Care and Use of Laboratory Animals (NIH Publication No. 8023, revised 1978) and were approved by the Institutional Animal Care and Use Committee at Anhui University of Science and Technology.

## 3 Results

### 3.1 Vitamin D3 supplementation accelerates the rate of weight gain in nicotine mice during the exposure

**Figure 1:**
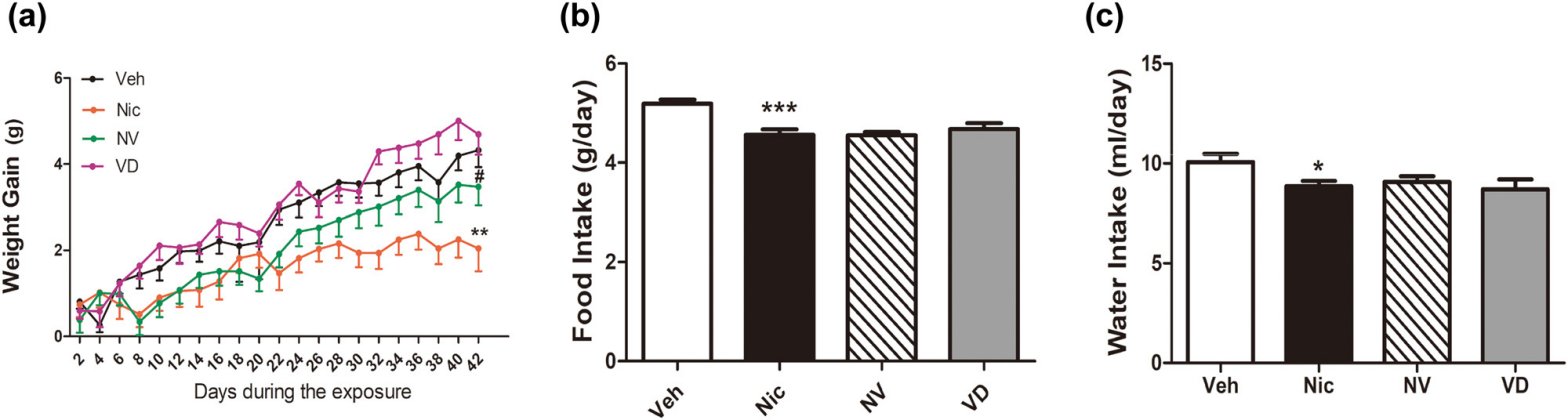
Vitamin D3-supplemented diet increases the weight gain in nicotine exposed-mice. Mice were monitored for their weight changes every 2 days (a), food intake (b) every 3 days, and water intake (c) daily during nicotine exposure. The duration of all treatments was 6 weeks. The weight gain in nicotine mice was slower than vehicle mice, but vitamin D3 reversed it (*n* = 16). Data were expressed as mean ± SEM. Compared with the vehicle group, *P < 0.05, **P < 0.01; compared with the Nic group, ^#^ *P* < 0.05, ^##^ *P* < 0.01.

To explore the effect of vitamin D3 supplementation on nicotine-exposure mice, weight gain, food intake, and water intake were regularly monitored. The rate of weight gain in the nicotine exposure mice was lower than the one received only vehicle treatment [(2.05 ± 2.13) g, (4.32 ± 1.57) g, *t* (15) = 3.63, *P* < 0.01; Figure 1]. Nicotine-exposed mice increased their weight in a lower degree, whereas mice supplemented with vitamin D3 offset this effect (Figure 1a). Moreover, after supplemented with vitamin D3, the rate of weight gain increased, compared with the mice that treated with nicotine only [(3.47 ± 1.74) g, (2.05 ± 2.13) g, *t* (15) = 3.27, *P* < 0.05; Figure 1a]. However, supplemented with vitamin D3 had little effect on the food and water intake during the exposure period, compared with nicotine-treated mice (Figure 1b and c).

### 3.2 Supplemented with Vitamin D3 ameliorates anxiety-like behavior induced by nicotine withdrawal

**Figure 2:**
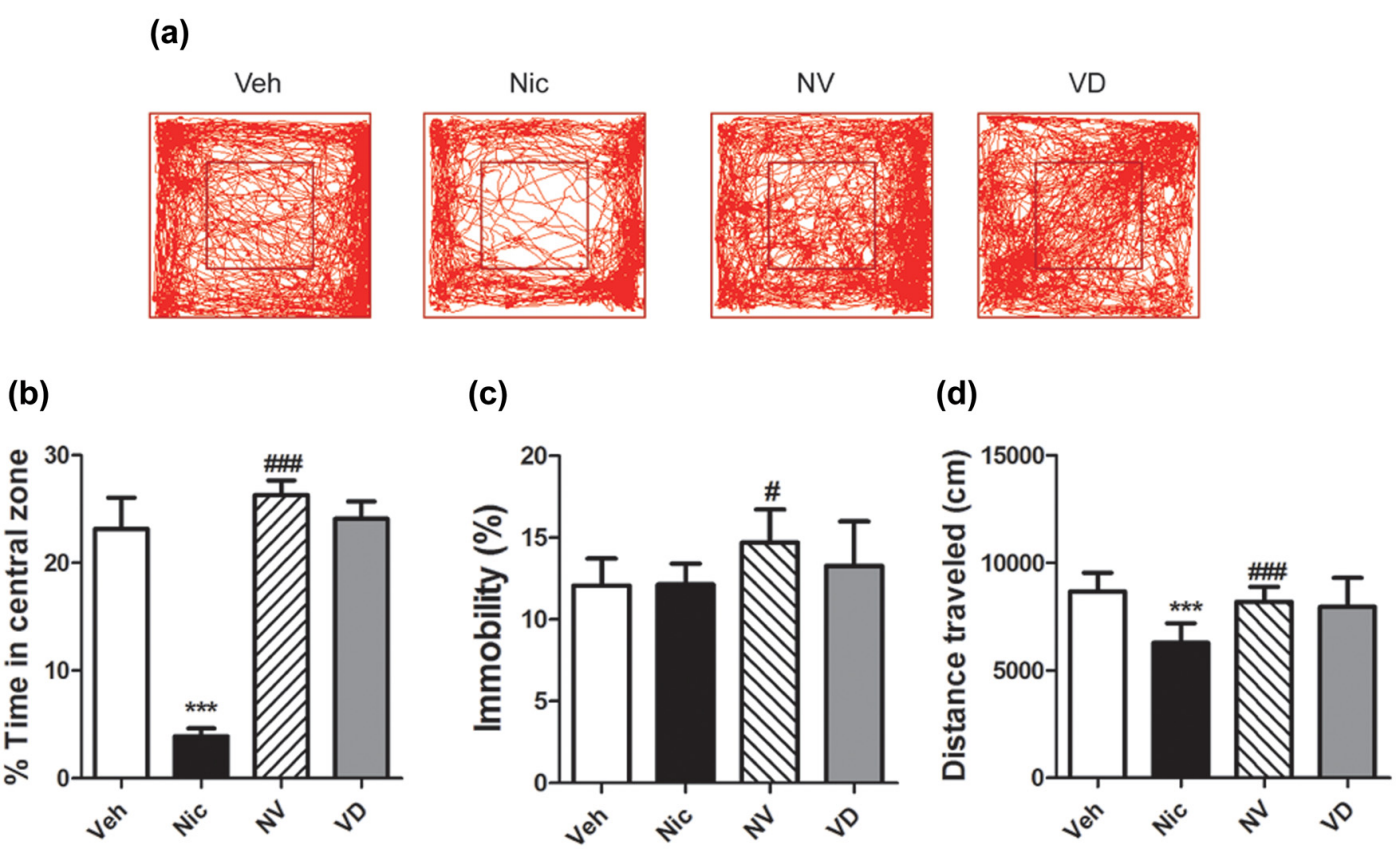
Vitamin D3 supplement in the diet alters exploration behavior in mice during spontaneous withdrawal. (a) Movement track within 30 min during the open-field test (*n* = 16). Nicotine-treated mice spent less time in the central zone and decrease total distance traveled relative to the vehicle one, while mice receiving vitamin D3-supplemented diet used much more time in the central area (*n* = 16) (b), increase immobility (*n* = 16) (c), and total distance traveled (*n* = 16) (d). Data were expressed as mean ± SEM. Compared with the vehicle group *P < 0.05, **P < 0.01; compared with the Nic group, ^#^ *P* < 0.05, ^##^ *P* < 0.01.

OFT showed that nicotine exposure mice performed the anxiety-like behavior because of spontaneous nicotine withdrawal, which showed less time in the central zone (*t* (15) = 21.06, *P* < 0.001; Figure 2b) and fewer traveled distances (movement track, Figure 2a; *t* (15) = 7.35, *P* < 0.001; Figure 2d) than the vehicle ones. In contrast, mice treated with vitamin D3 counteracted the above performance, with the duration (*t* (15) = 24.49, *P* < 0.001; Figure 2b) and traveled distance (movement track, Figure 2a; *t* (15) = 4.21, *P* < 0.001; Figure 2d) in the central zone significantly longer than the nicotine-exposure mice. There was no difference in immobility between nicotine mice and vehicle mice (*t* (15) = 0.05, *P* > 0.05; Figure 2c).

**Figure 3:**
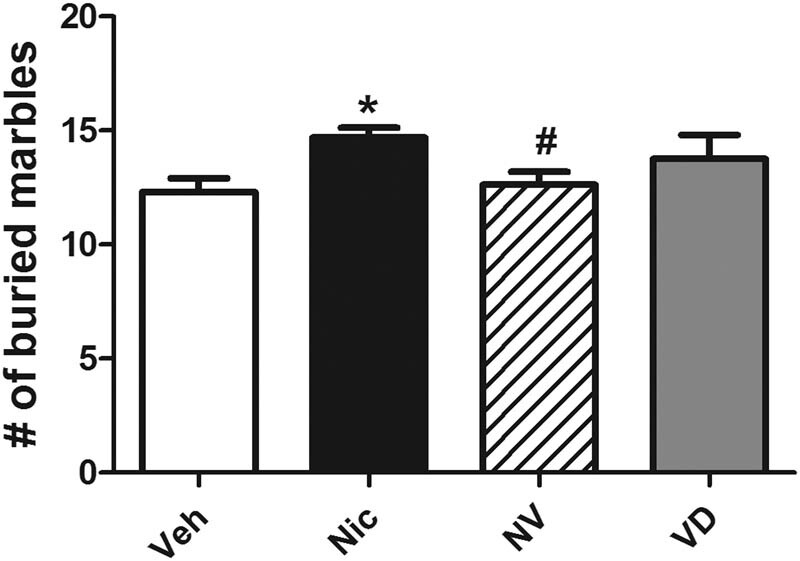
Mice fed with the vitamin D3-supplemented present a decrease in “threats” buried during spontaneous withdrawal. Data were expressed as mean ± SEM (*n* = 16). Compared with the vehicle group *P < 0.05, **P < 0.01; compared with the Nic group, ^#^ *P* < 0.05, ^##^ *P* < 0.01.

To detect the anxiety-related behavior multiply, the MBT was carried out 12 h after the end of the OFT. Nicotine-exposed mice have more buried marbles during spontaneous nicotine withdrawal which is considered a performance of anxiety-like behavior, compared to the mice that received only vehicle treatment (*t* (15) = 2.93, *P* < 0.05; Figure 3). By contrary, supplemented with vitamin D3, the anxiety-related behavior was obviously relieved between the vitamin D3-treated mice and the nicotine-exposure mice, with the significantly fewer numbers of buried marble (*t* (15) = 2.54, *P* < 0.05; Figure 3).

### 3.3 The hippocampal upregulation of NR2A level induced by nicotine withdrawal is corrected in mice fed the vitamin D3-supplemented diet

**Figure 4:**
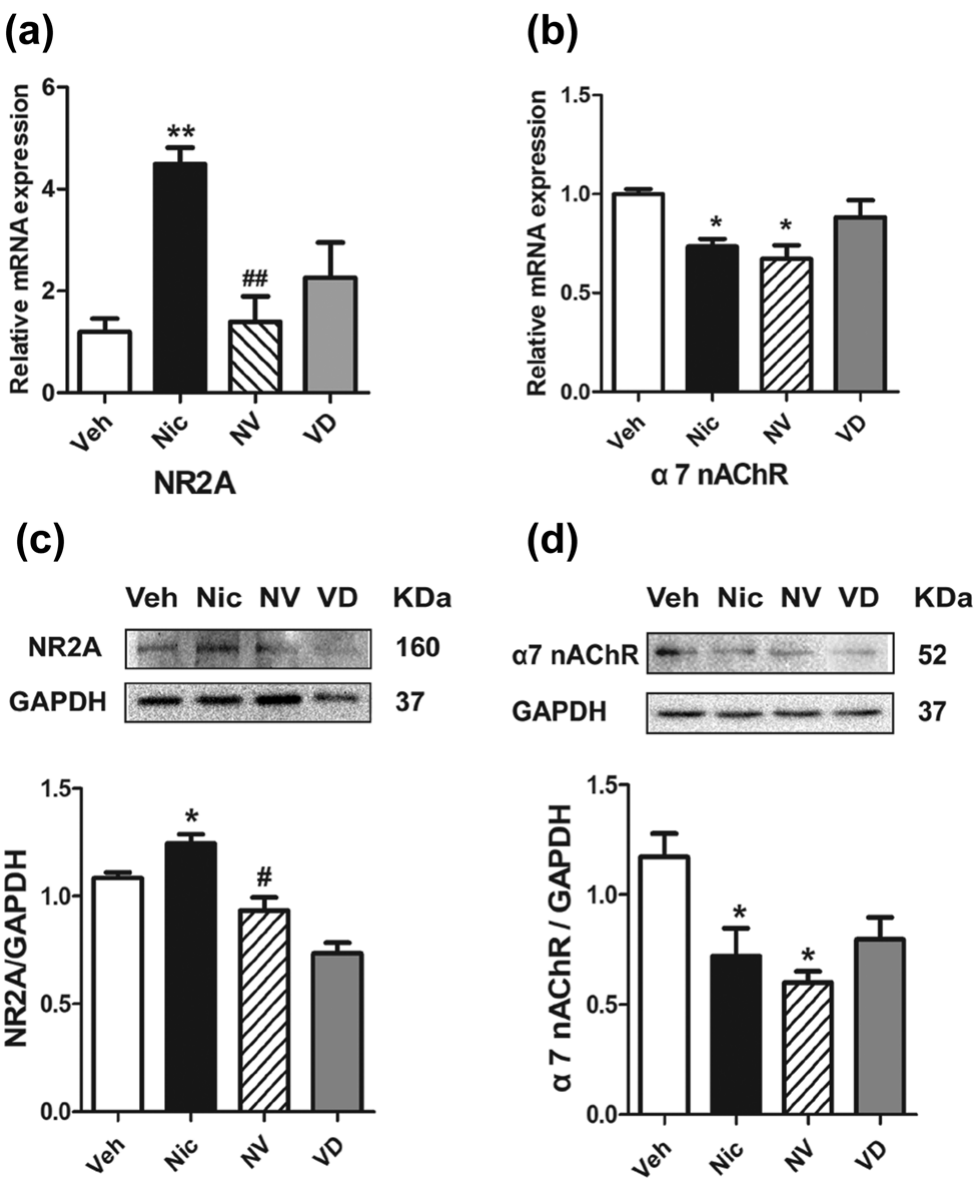
The hippocampal upregulation of NR2A level induced by nicotine withdrawal is corrected in mice fed with the vitamin D3-supplemented diet. NR2A mRNA level (a) and protein level (c) were significantly decreased after receiving vitamin D3 supplement, compared to nicotine-treated mice (*n* = 8). There was a slight but significant decrease in hippocampal α7 nAChR in the nicotine-treated and vitamin D3-supplemented mice, compared with the vehicle group (*n* = 8), both in mRNA expression (b) and in protein level (d). GAPDH was used as a reference protein (*n* = 8). Data were expressed as mean ± SEM. Compared with the vehicle group *P < 0.05, **P < 0.01; compared with the Nic group, ^#^ *P* < 0.05, ^##^ *P* < 0.01.

The expressions of NR2A and α7 nAChR in the hippocampus were examined with WB and QPCR, respectively. The nicotine-treated mice presented an increase in the relative mRNA expression of hippocampal NR2A (*t* (7) = 4.95, *P* < 0.01; Figure 4a) and protein bands (*t* (7) = 3.02, *P* < 0.05; Figure 4c) as compared to the vehicle mice during spontaneous withdrawal. By contrast, a significant decrease in NR2A protein level (*t* (7) = 6.13, *P* < 0.05; Figure 4c) and the relative mRNA expression (*t* (7) = 4.66, *P* < 0.01; Figure 4a) were observed in the vitamin D3-treated mice, compared with the nicotine-exposed mice.

To detect the underlying mechanism from different perspective, the hippocampal α7 nAChR protein and mRNA were tested. Interestingly, α7 nAChR protein level (*t* (7) = 3.15, *t* = 3.99, *P* < 0.05; Figure 4d) and relative mRNA expression (*t* (7) = 3.15, *t* = 3.99, *P* < 0.05; Figure 4b) were significantly declined in nicotine exposure hippocampus. On the contrary, there was no difference in the protein and relative mRNA expression of α7 nAChR in hippocampus between the vitamin D3-treated mice and only nicotine-exposed mice.

### 3.4 Vitamin D3 supplemented in diet increases the 25(OH)D3 serum concentration, while there is a slight decrease in the cotinine level as compared with only nicotine-exposed mice

For quantitative analysis of indicators concerning nicotine and vitamin D3, the serum concentration of 25(OH)D3 and cotinine were measured by ELISA. Serum cotinine levels in the nicotine mice were significantly higher than vehicle mice (*t* (3) = 7.21, *P* < 0.001; Table 2), which had at least 300 ng/mL. Interestingly, cotinine serum concentration was only slightly declined between the vitamin D3-treated mice and the nicotine-exposure mice (*t* (3) = 0.87, *P* > 0.05; Table 2). After supplemented with vitamin D3, mice presented a significant increase in the 25(OH)D3 serum level (22.90 ± 3.45, 43.37 ± 3.95 ng/mL; *t* (3) = 3.46, *P* < 0.05; Table 2) as compared to the nicotine exposure only.

**Table 2:** Expression of 25(OH)D3 and cotinine in the serum by ELISA (ng/mL)

Group	25(OH)D3	Cotinine
Vehicle	24.93 ± 1.83	4.54 ± 0.56
Nic	22.90 ± 3.45	766.33 ± 2.18***
NV	43.37 ± 3.95^#^	674.33 ± 0.53
VD	42.20 ± 4.90	3.68 ± 1.55

ELISA detected a significant increase in 25(OH)D3 in vitamin D3-supplemented mice whereas were only slightly decreased for cotinine secretion (*n* = 3). Data were expressed as mean ± SEM. Compared with the vehicle group *P < 0.05, **P < 0.01, ***P < 0.001; compared with the Nic group, ^#^ *P* < 0.05, ^##^ *P* < 0.01.

## 4 Discussion

In this study, the effect of vitamin D3 supplementation in diet that reduces nicotine withdrawal-associated anxiety-like behavior was investigated. It was found that there is a dramatic difference in behavioral and biological responses to nicotine withdrawal between mice with and without vitamin D3-supplemented diet. When anxiety-like behavior occurs during withdrawal, nicotine-exposed mice showed decreased time and reduced traveled distances in the central area, while vitamin D3-supplemented ones spent more time in the center zone of the arena and presented increased overall locomotion. Furthermore, vitamin D3 diet intervention downregulated the expression level of hippocampal NR2A, which had been elevated in nicotine-exposed mice.

Vitamin D3, a precursor of 1,25(OH)_2_D3, is related to the metabolism of neurons in the brain [18]. Many biological effects of vitamin D in the nervous system are reported, including the downregulation of L-type calcium channel expression in hippocampal neurons, the inhibition of induced nitric oxide synthase synthesis, coupled with the increase in glutathione levels and so on, indicating that vitamin D plays an important role in brain detoxification and neuroprotection [10,19,20]. Calcium ions enter the cell through the NMDA receptor in limbic areas, such as the hippocampus, which in turn activates neuronal nitric oxide synthase leading to the synthesis of NO [21,22]. Liu et al. found that acupuncture attenuates nicotine withdrawal-induced anxiety through the inhibition of the NMDA receptor/nitric oxide synthase (NMDAR/NOS) pathway [23]. First, our data revealed that vitamin D3 ameliorated the anxiety-like behaviors during nicotine withdrawal and downregulated the expression of NR2A in the hippocampus, which may be related to the inhibition of the NMDAR/NOS pathway.

Vitamin D has been investigated for the treatment of anxiety disorders because of its pharmacological effects such as antioxidant and anti-inflammatory properties [24,25,26]. These findings suggest that 1,25(OH)_2_D3 and its pharmacological analogues have potential value in the treatment of neurodegenerative, neuron-immune diseases, and anxiety. In recent years, vitamin D has been shown to have certain anti-anxiety and anti-depressant effects [26]. Long-term administration of vitamin D3 performed anti-anxiety effects in both ovariectomized female rats and ovariectomized rats treated with 17β-estradiol [24,25], and its mechanism may be related to the increased expression of dopamine and serotonin in hippocampus [25].

Here we show that vitamin D3 downregulated the expression of NR2A, which may be related to the amelioration of anxiety-like behaviors. After exposure with nicotine for 42 days, the serum of cotinine concentration was as high as 766 ng/mL, reaching a dose of nicotine addiction (300 ng/mL), and which was higher than that of heavy smokers (>30 cigarettes per day) described previously [17]. Therefore, this oral dosage of nicotine is equivalent to smoking more than 30 cigarettes a day. After dietary vitamin D3 supplement, serum 25(OH)D3 level increased in nicotine mice, but the cotinine level is not impacted in vitamin D3 supplemented mice group as described in Table 2. We speculate that the improvement of less anxiety-like behavior during nicotine withdrawal is related to vitamin D3 supplementation, which would not affect the process of nicotine metabolism. Moreover, during nicotine withdrawal, the nicotine exposure mice were less exploratory in the OFT, and the number of “threats” buried in the MBT increased, indicating the presence of anxiety-like behaviors. These results are consistent with the findings of Lee et al. that after nicotine withdrawal, the number of marble buried in mice increased and the duration in the center of OFT was shortened [27]. Importantly, after vitamin D3 dietary intervention, anxiety-like behaviors were alleviated.

Massive studies have shown that NMDA receptors in the hippocampus are involved in neurobiological processes which are related to emotions including fear, anxiety, and depression [5,6]. The activation and expression of NMDA receptors are involved in the anxiety-like behavior induced by nicotine withdrawal. After administrated with nicotine, the expression of NMDA in the central amygdala of rats was upregulated, showing anxiety-like behavior [8]. Previous studies have also shown that NMDA receptor antagonists relieved anxiety symptoms by blocking the transmission of glutamatergic signals [12].

Most NMDA receptors contain two obligate GluN1 subunits and two GluN2 subunits, of which there are four types (2A-D), with NR2A and NR2B being predominant in the forebrain. Previously, the administration of nicotine *in vivo* increases NR2B-containing NMDA receptors (NR2B-NMDARs) responses in hippocampal CA1 pyramidal cells, which are a point of convergence of cholinergic and glutamatergic pathways involved in learning and memory [28]. There is no research, though, on the direct relationship between NR2A and anxiety symptom after nicotine withdrawal. In addition, NR2A determines the biophysical properties of NMDA receptors in the forebrain (including hippocampus) and downstream signal transduction [29], regulates the downward transmission of glutamatergic signals, and then causes anxiety-like behavior during nicotine withdrawal. Therefore, we have chosen to investigate the gene and protein expression of NR2A.

As one of the most abundant nAChRs in the nervous system, α7 nAChR is highly expressed in hippocampus, cortex, and several subcortical limbic regions [30], and closely related to cognition and reward pathways. The α7 subunit of the nAChRs has been understudied in nicotine withdrawal, even though it is expressed in brain regions important for drug reward [31]. Recently, Galvez et al. found that nicotine-induced depolarization of hippocampal CA1 neurons in rat activated the GluN2A and desensitized α7 nAChR [32]. Desensitization refers to signal termination and decreased response that occurs with repeated or chronic exposure to agonist. Mechanistically, desensitization can be divided into receptor downregulation (i.e., reduction in total receptor number) by degradation of the ligand–receptor complex after endocytosis; receptor inactivation by binding of protein beta-arrestin after phosphorylation; receptor low sensitivity to signal response; a negative feedback by producing inhibitory protein in downstream signals; and receptor internalization by endocytosis. Similarly, our data indicate that in the hippocampus of nicotine withdrawal mice, the expression of α7 nAChR was significantly decreased and NR2A was increased in both Nic and NV groups with nicotine exposure. After vitamin D3 treatment, however, the level of α7 nAChR in nicotine mice did not change significantly. α7 nAChR desensitization may contribute to its decrease in nicotine exposed hippocampus, which has been reported previously [31,32].

In the present study, we used chronic nicotine exposure mouse model, investigated the anti-anxiety effect of vitamin D3, and associated downstream signals during nicotine withdrawal. Our data directly support that the downregulation of hippocampal NR2A expression with vitamin D3 supplementation in diet is related to the ameliorated anxiety-like behavior. Anxiety is common in a variety of diseases and population. As a new type of anti-anxiety nutrient, the specific molecular mechanism of vitamin D3 is still unclear. Owing to the advantages of easy access and minimal side effects, however, vitamin D3 may provide an effective, safety, and tolerability of medications for the amelioration of anxiety-like behavior assisting long-term tobacco smoking cessation in people who smoke cigarettes.
